# 2-({[(Pyridin-1-ium-2-ylmeth­yl)carbamo­yl]form­amido}­meth­yl)pyridin-1-ium bis­(3,5-di­carb­oxy­benzoate): crystal structure and Hirshfeld surface analysis

**DOI:** 10.1107/S2056989016000980

**Published:** 2016-01-27

**Authors:** Mukesh M. Jotani, Sabrina Syed, Siti Nadiah Abdul Halim, Edward R. T. Tiekink

**Affiliations:** a Department of Physics, Bhavan’s Sheth R. A. College of Science, Ahmedabad, Gujarat 380 001, India; b Department of Chemistry, University of Malaya, 50603 Kuala Lumpur, Malaysia; c Centre for Crystalline Materials, Faculty of Science and Technology, Sunway University, 47500 Bandar Sunway, Selangor Darul Ehsan, Malaysia

**Keywords:** crystal structure, salt, hydrogen bonding, carboxyl­ate, di­amide, Hirshfeld surface analysis

## Abstract

The asymmetric unit of the title salt, C_14_H_16_N_4_O_2_^2+^·2C_9_H_5_O_6_^−^, comprises half a dication, being located about a centre of inversion, and one anion, in a general position. The central C_4_N_2_O_2_ group of atoms in the dication are almost planar (r.m.s. deviation = 0.009 Å), and the carbonyl groups lie in an *anti* disposition to enable the formation of intra­molecular amide-N—H⋯O(carbon­yl) hydrogen bonds. To a first approximation, the pyridinium and amide N atoms lie to the same side of the mol­ecule [N_py_—C—C—N_amide_ torsion angle = 34.8 (2)°], and the *anti* pyridinium rings are approximately perpendicular to the central part of the mol­ecule [dihedral angle = 68.21 (8)°]. In the anion, one carboxyl­ate group is almost coplanar with the ring to which it is connected [C_ben_—C_ben_—C_q_—O torsion angle = 2.0 (3)°], whereas the other carboxyl­ate and carb­oxy­lic acid groups are twisted out of the plane [torsion angles = 16.4 (3) and 15.3 (3)°, respectively]. In the crystal, anions assemble into layers parallel to (10-4) *via* hy­droxy-O—H⋯O(carbon­yl) and charge-assisted hy­droxy-O—H⋯O(carboxyl­ate) hydrogen bonds. The dications are linked into supra­molecular tapes by amide-N—H⋯O(amide) hydrogen bonds, and thread through the voids in the anionic layers, being connected by charge-assisted pyridinium-N—O(carboxyl­ate) hydrogen bonds, so that a three-dimensional architecture ensues. An analysis of the Hirshfeld surface points to the importance of O—H⋯O hydrogen bonding in the crystal structure.

## Abstract 2

The crystal structure of the title salt comprises supra­molecular tapes of dications arising from amide-N—H⋯O(amide) hydrogen bonds which thread through supra­molecular layers of anions connected *via* hy­droxy-O—H⋯O(carbon­yl) and charge-assisted hy­droxy-O—H⋯O(carboxyl­ate) hydrogen bonds.

## Chemical context   

Of the isomeric *N,N′*-bis­(pyridin-*n*-ylmeth­yl)ethanedi­amides, *n* = 2, 3 or 4, the mol­ecule with *n* = 2 appears to have attracted the least attention in co-crystallization studies; for the chemical structure of the diprotonated form of the *n* = 2 isomer see Scheme 1. By contrast, the *n* = 3 and 4 mol­ecules have attracted inter­est from the crystal engineering community in terms of their ability to form co-crystals with iodo-containing species leading to aggregates featuring N⋯I halogen bonding (Goroff *et al.*, 2005 ▸; Jin *et al.*, 2013 ▸) as well as carb­oxy­lic acids (Nguyen *et al.*, 2001 ▸). It is the latter that has formed the focus of our inter­est in co-crystallization experiments of these mol­ecules which has led to the characterization of both co-crystals (Arman, Kaulgud *et al.*, 2012 ▸; Arman, Miller *et al.*, 2012 ▸) and salts (Arman *et al.*, 2013 ▸). It was during the course of recent studies in this area (Syed *et al.*, 2016 ▸) that the title salt was isolated from the 1:1 co-crystallization experiment between the *n* = 2 isomer and trimesic acid. The crystal and mol­ecular structures as well as a Hirshfeld surface analysis of this salt is described herein.
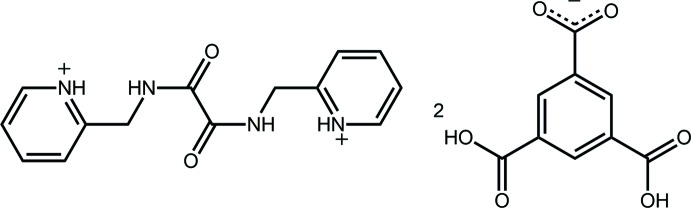


## Structural commentary   

**Figure 1:**
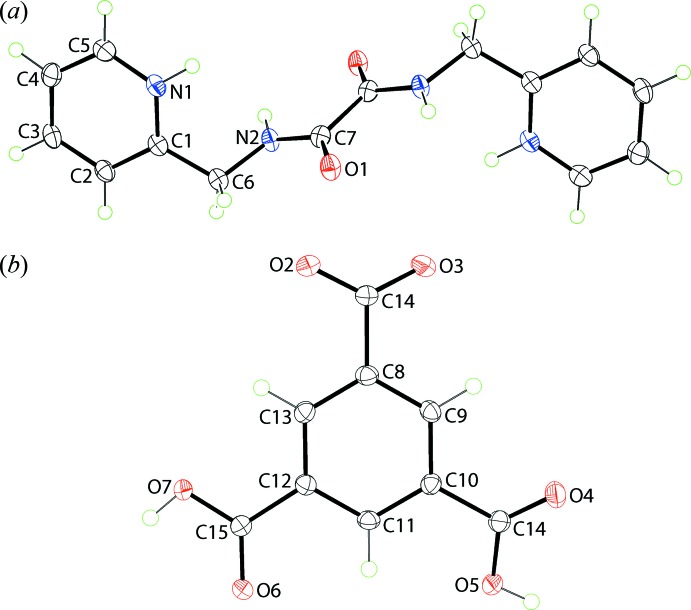
The mol­ecular structures of the ions comprising the title salt, showing the atom-labelling scheme and displacement ellipsoids at the 50% probability level: (*a*) 2-({[(pyridin-1-ium-2-ylmeth­yl)carbamo­yl]formamido}­meth­yl)pyridin-1-ium, and (*b*) 3,5-di­carb­oxy­benzoate; unlabelled atoms are related by the symmetry operation −*x*, 1 − *y*, 1 − *z*.

The title salt, Fig. 1 ▸, was prepared from the 1:1 reaction of trimesic acid and *N,N′*-bis­(pyridin-2-ylmeth­yl)ethanedi­amide conducted in ethanol. The harvested crystals were shown by crystallography to comprise (2-pyridinium)CH_2_N(H)C(=O)C(=O)CH_2_N(H)(2–pyridinium) dications and 3,5-di­carb­oxy­benzoate anions in the ratio 1:2; as the dication is located about a centre of inversion, one anion is found in the asymmetric unit. The confirmation for the transfer of protons during the co-crystallization experiment is found in (i) the pattern of hydrogen-bonding inter­actions as discussed in *Supra­molecular features*, and (ii) the geometric characteristics of the ions. Thus, the C—N—C angle in the pyridyl ring has expanded by over 3° *cf*. that found in the only neutral form of *N,N′*-bis­(pyridin-2-ylmeth­yl)ethanedi­amide characterized crystallographically in an all-organic mol­ecule, *i.e*. in a 1:2 co-crystal with 2-amino­benzoic acid (Arman, Miller *et al.*, 2012 ▸), Table 1 ▸. The observed angle is in agreement with the sole example of a diprotonated form of the mol­ecule, *i.e*. in a 1:2 salt with 2,6-di­nitro­benzoate (Arman *et al.*, 2013 ▸), Table 1 ▸. Further, the experimental equivalence of the C14—O2, O3 bond lengths, *i.e*. 1.259 (2) and 1.250 (2) Å is consistent with deprotonation and the formation of a carboxyl­ate group, and contrasts the great disparity in the C15—O4, O5 [1.206 (2) and 1.320 (2) Å] and C16—O6, O7 [1.229 (2) and 1.315 (2) Å] bond lengths.

**Figure 2:**
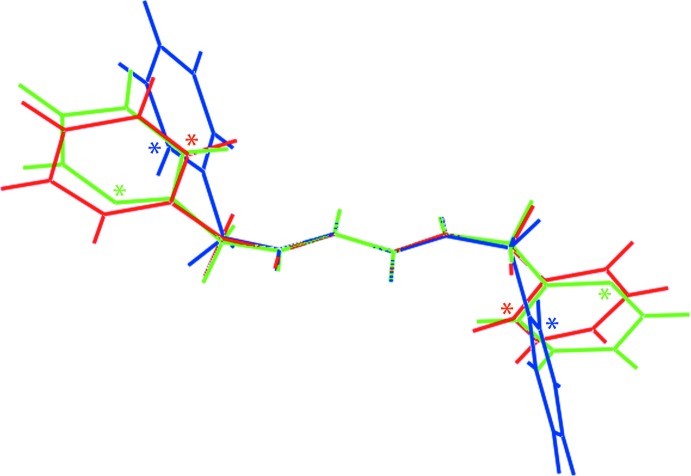
Overlay diagram of the dication in the title compound (red image), the neutral mol­ecule in its co-crystal (green), and dication in the literature salt (blue). The mol­ecules have been overlapped so that the O=C—C=O residues are coincident. The ring N atoms are indicated by an asterisk.

In the dication, the central C_4_N_2_O_2_ chromophore is almost planar, having an r.m.s. deviation of 0.009 Å and, from symmetry, the carbonyl groups are *anti*. An intra­molecular amide-N—H⋯O(carbon­yl) hydrogen bond is noted, Table 2 ▸. The pyridinium-N1 and amide-N2 atoms are approximately *syn* as seen in the value of the N1—C1—C6—N2 torsion angle of 34.8 (2)°. This planarity does not extend to the terminal pyridinium rings which are approximately perpendicular to and lying to either side of the central chromophore, forming dihedral angles of 68.21 (8)°. The central C7—C7^i^ bond length of 1.538 (4) Å is considered long for a C—C bond involving *sp* ^2^-hybridized atoms (Spek, 2009 ▸). Geometric data for the two previously characterized mol­ecules (Arman, Miller *et al.*, 2012 ▸; Arman *et al.*, 2013 ▸) related to the dication are collected in Table 1 ▸. To a first approximation, the three mol­ecules present the same features as described above with the notable exception of the relative disposition of the pyridinium-N1 and amide-N2 atoms. Thus, in the neutral form of the mol­ecule, these are *anti*, the N1—C1—C6—N2 torsion angle being 165.01 (10) Å, and almost perpendicular in the salt, with N1—C1—C6—N2 being 73.84 (15)°. These differences are highlighted in the overlay diagram shown in Fig. 2 ▸.

In the anion, the C13—C8—C14—O2 and C9—C10—C15—O4 torsion angles of 15.3 (3) and 16.4 (3)°, respectively, indicate twisted conformations between these residues and the ring to which they are attached whereas the C11—C12—C16—O6 torsion angle of 2.0 (3)° shows this carb­oxy­lic acid group to be co-planar with the ring. The conformational flexibility in 3,5-di­carb­oxy­benzoate anions is well illustrated in arguably the four most closely related structures in the crystallographic literature (Groom & Allen, 2014 ▸), identified from approximately 35 organic salts containing this anion. Referring to Scheme 2, the most closely related structure features the dication C_I with two protonated pyridyl N atoms (Santra *et al.*, 2009 ▸). Here, with two crystallographically independent anions, twists are noted from the mean-plane data collated in Table 3 ▸. For one anion, all groups are twisted out of the least-squares plane through the benzene ring but, in the second anion, the carboxyl­ate group is effectively co-planar with the ring with up to a large twist noted for one of the carb­oxy­lic acid groups. In the other example with a diprotonated cation, C_II (Singh *et al.*, 2015 ▸), both independent anions exhibit twists of less than 8° with all three residues effectively co-planar in one of the anions. In the example with a single protonated pyridyl residue, C_III (Ferguson *et al.*, 1998 ▸), twists are evident for one of the carb­oxy­lic acid groups and for the carboxyl­ate but, the second carb­oxy­lic acid residue is effectively co-planar. Finally, in the mono-protonated species related to C_I, *i.e*. C_IV (Basu *et al.*, 2009 ▸), twists are evident for all groups with the maximum twists observed in the series for the carboxyl­ate residue, *i.e*. 25.13 (10)°, and for one of the carb­oxy­lic acid groups, *i.e*. 22.50 (10)°.
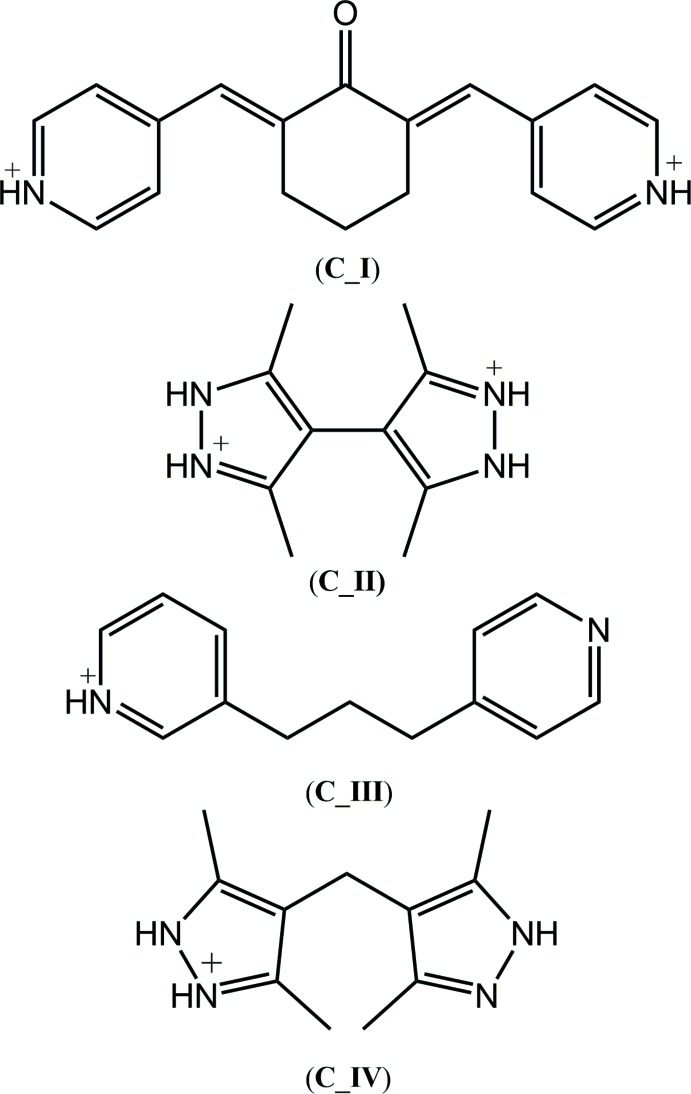


## Supra­molecular features   

**Figure 3:**
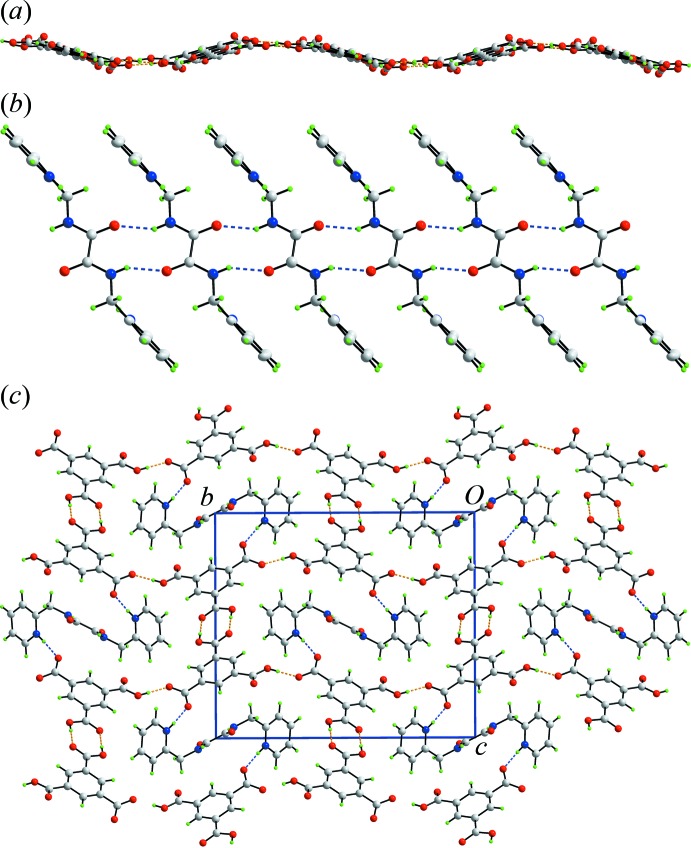
Mol­ecular packing in the title salt: (*a*) supra­molecular layers mediated by O—H⋯O hydrogen bonds, (*b*) supra­molecular tapes mediated by N—H⋯O hydrogen bonds, and (*c*) a view of the unit-cell contents shown in projection down the *a* axis, whereby the supra­molecular layers, illustrated in Fig. 3 ▸(*a*), are linked by charge-assisted N—H⋯O(carboxyl­ate) hydrogen bonds to consolidate a three-dimensional architecture. The O—H⋯O and N—H⋯O hydrogen bonds are shown as orange and blue dashed lines, respectively.

The mol­ecular packing may be conveniently described in terms of O—H⋯O hydrogen bonding to define an anionic network which is connected into a three-dimensional architecture by N—H⋯O hydrogen bonds; Table 2 ▸ collates geometric data for the inter­molecular inter­actions discussed in this section. Thus, centrosymmetrically related C—O6,O7 carb­oxy­lic acid groups associate *via* hy­droxy-O—H⋯O(carbon­yl) hydrogen bonds to form a familiar eight-membered {⋯HOCO}_2_ synthon. These are connected by charge-assisted hy­droxy-O—H⋯O(carboxyl­ate) hydrogen bonds that form *C*(8) chains. The result is a network of anions lying parallel to (10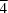) and having an undulating topology, Fig. 3 ▸ *a*. The dications also self-associate to form supra­molecular tapes *via C*(4) chains featuring pairs of amide-N—H⋯O(amide) hydrogen bonds and 10-membered {⋯HNC_2_O}_2_ synthons, Fig. 3 ▸ *b*. The tapes are aligned along the *a* axis and, in essence, thread through the voids in the anionic layers to form a three-dimensional architecture, Fig. 3 ▸ *c*. The links between the anionic layers and cationic tapes are hydrogen bonds of the type charge-assisted pyridinium-N—O(carboxyl­ate). In this scheme, no apparent role for the carbonyl-O4 atom is evident. However, this atoms accepts two C—H⋯O inter­actions from pyridyl- and methyl­ene-H to consolidate the mol­ecular packing. Additional stabilization is afforded by pyridyl-C—H⋯O(carboxyl­ate, carbon­yl) inter­actions, Table 2 ▸.

## Analysis of the Hirshfeld surfaces   

*Crystal Explorer* 3.1 (Wolff *et al.*, 2012 ▸) was used to generate Hirshfeld surfaces (Spackman & Jayatilaka, 2009 ▸) mapped over *d*_norm_, *d*_e_ and electrostatic potential for the title salt. The electrostatic potentials were calculated using *TONTO* (Spackman *et al.*, 2008 ▸; Jayatilaka *et al.*, 2005 ▸) integrated with *Crystal Explorer*, and mapped on the Hirshfeld surfaces using the STO-3G basis set at the Hartree–Fock level theory over the range ±0.25 au. The contact distances *d*_i_ and *d*_e_ from the Hirshfeld surface to the nearest atom inside and outside, respectively, enable the analysis of the inter­molecular inter­actions through the mapping of *d*_norm_. The combination of *d*_e_ and *d*_i_ in the form of two-dimensional fingerprint plots provides a summary of inter­molecular contacts in the crystal (Rohl *et al.*, 2008 ▸).

**Figure 4:**
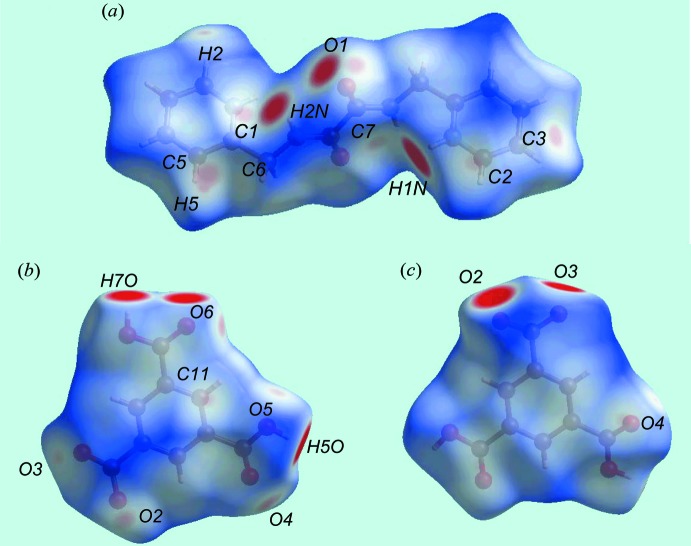
Views of the Hirshfeld surface mapped over *d*_norm_ in the title salt: (*a*) dication, (*b*) and (*c*) anion.

**Figure 5:**
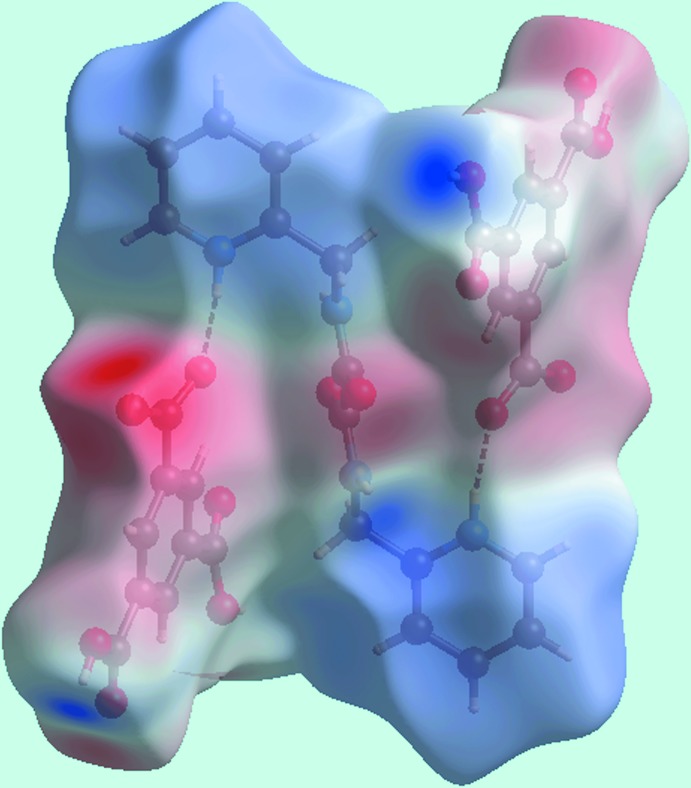
View of the Hirshfeld surface mapped over the calculated electrostatic potential the tri-ion aggregate in the title salt.

**Figure 6:**
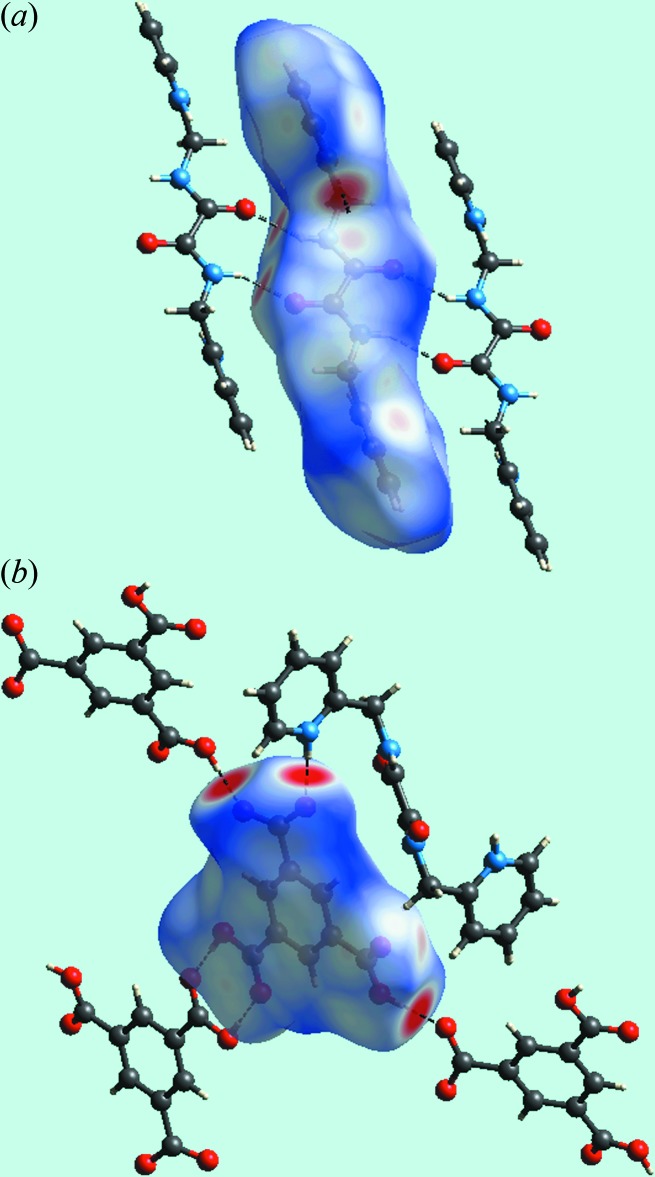
Views of the Hirshfeld surfaces mapped over *d*_norm_ in the title salt emphasizing the inter­actions between (*a*) dianions and (*b*) the environment about the anion.

Views of the Hirshfeld surface mapped over *d*_norm_ in the title salt are given in Fig. 4 ▸. The formation of charge-assisted hydroxyl-O—H⋯O(carboxyl­ate) and pyridinium-N—H⋯O(carboxyl­ate) hydrogen bonds in the crystal appear as distinct dark-red spots near the respective donor and acceptor atoms. In Fig. 5 ▸, the blue and red colouration are the corres­ponding regions on the surface mapped over the electrostatic potential. The dark-red spots on the Hirshfeld surface of the dication corresponds to a pair of amide-N—H⋯O(amide) hydrogen bonds leading to the supra­molecular tape. Inter­molecular C—H⋯O and N—H⋯O inter­actions, representing weak hydrogen bonds over and above those discussed above in *Supra­molecular features*, result in light-red spots near some of the carbon, nitro­gen and oxygen atoms, Fig. 4 ▸. Hence, the contribution to the surface from these inter­actions involve not only O⋯H/H⋯O contacts but also C⋯O/O⋯C and N⋯O/O⋯N contacts, Table 4 ▸. The relative contributions of the different contacts to the Hirshfeld surfaces are collated in Table 5 ▸ for the entire structure and also delineated for the dication and anion. The linkage of ions through the formation of hydrogen bonds is illustrated in Fig. 6 ▸.

**Figure 7:**
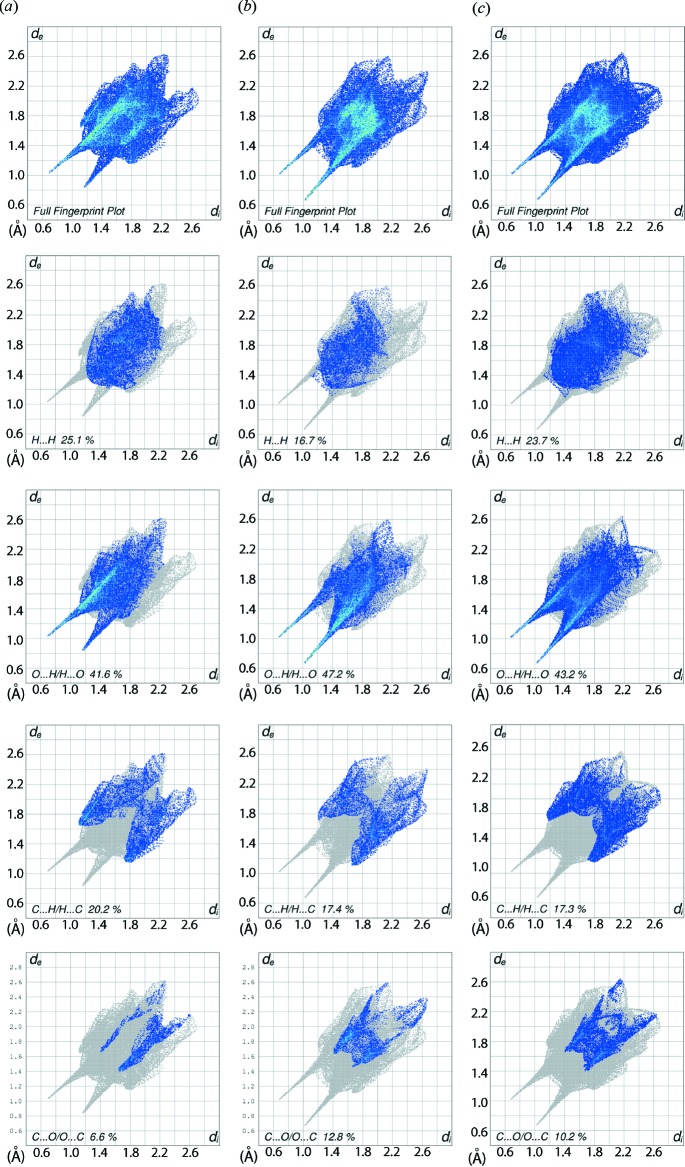
The two-dimensional fingerprint plots for the title salt: (*a*) dication, (*b*) anion, and (*c*) full structure, showing contributions from different contacts, *i.e*. H⋯H, O⋯H/H⋯O, C⋯H/H⋯C, and C⋯O/O⋯C.

The overall two-dimensional fingerprint plot (FP) of the salt together with those of the dication and anion, and FP’s delineated into H⋯H, O⋯H/H⋯O, C⋯H/H⋯C and C⋯O/O⋯C contacts are illustrated in Fig. 7 ▸. The O⋯H/H⋯O contacts have the largest overall contribution to the Hirshfeld surface, *i.e*. 43.2%, and these inter­actions dominate in the crystal structure. The prominent spike with green points appearing in the lower left region in the FP for the anion at *d*_e_ + *d*_i_ ∼ 1.7 Å has a major contribution, *i.e*. 47.2%, from O⋯H contacts; the spike at the same *d*_e_ + *d*_i_ distance is due to a small contribution, 10.0%, from H⋯O contacts. The different contributions from O⋯H and H⋯O contacts to the Hirshfeld surface of the dication, *i.e*. 6.8 and 34.8%, respectively, lead to asymmetric peaks at *d*_e_ + *d*_i_ ∼ 1.8 and 2.0 Å, respectively, indicating the varying strength of these inter­actions. However, the overall FP of the salt delineated into O⋯H/H⋯O contacts shows a symmetric pair of spikes at *d*_e_ + *d*_i_ ∼ 1.7 Å with nearly equal contributions from O⋯H and H⋯O contacts. A smaller contribution is made by the H⋯H contacts, Table 1 ▸, and these appear as the scattered points without a distinct peak, Fig. 7 ▸. The presence of short inter­atomic C⋯H/H⋯C contacts, Table 4 ▸, result in a 17.3% overall contribution to the surface, although there are no C—H⋯π contacts within the acceptance distance criteria for such inter­actions (Spek, 2009 ▸). These are represented by a pair of symmetrical wings at *d*_e_ + *d*_i_ ∼ 2.9 Å in the FP plot, Fig. 7 ▸. The contribution from C⋯O/O⋯C contacts to the Hirshfeld surface is also evident from the presence of inter­molecular C—H⋯O inter­actions as well as short inter­atomic C⋯O/O⋯C contact, Table 4 ▸. These appear as cross-over wings in the (*d*_e_, *d*_i_) region between 1.7 and 2.7 Å. A small but significant contribution to the Hirshfeld surface of the dication due to N⋯O/O⋯N contacts is the result of inter­molecular amide-N—H⋯O(amide) inter­actions.

The inter­molecular inter­actions were further analysed using a recently reported descriptor, the enrichment ratio, ER (Jelsch *et al.*, 2014 ▸), which is based on Hirshfeld surface analysis and gives an indication of the relative likelihood of specific inter­molecular inter­actions to form; the calculated ratios are given in Table 6 ▸. The relatively poor content of hydrogen atoms in the salt and the involvements of many hydrogen atoms in the inter­molecular inter­actions, as discussed above, reduces the ER value of non-bonded H⋯H contacts to a value less unity, *i.e*. 0.8, due to a 23.7% contribution from the 54.5% available Hirshfeld surface and anti­cipated 29.7% random contacts. The ER value of 1.4 corresponding to O⋯H/H⋯O contacts results from a relatively high 43.2% contribution by O—H⋯O, N—H⋯O and C—H⋯O inter­actions. The carbon and oxygen atoms involved in the inter­molecular C—H⋯O inter­actions and short inter C⋯O/O⋯C contacts are at distances shorter than the sum of their respective van der Waals radii, hence they also have a high formation propensity, so the ER value is > 1. The C⋯H/H⋯C contacts in the crystal are enriched due to the poor nitro­gen content and the presence of short inter­atomic C⋯H/H⋯C contacts so the ratio is close to unity, *i.e*. 0.99. Finally, the ER value of 1.68 corresponding to N⋯O/O⋯N contacts for the surface of dication is the result of the charge-assisted N—H⋯O inter­actions consistent with their high propensity to form.

## Database survey   

As mentioned in the *Chemical context*, *N,N′*-bis­(pyridin-2-ylmeth­yl)ethanedi­amide (LH_2_), has not been as well studied as the *n* = 3 and 4 isomers. This notwithstanding, the coordin­ation chemistry of LH_2_ is more advanced and diverse. Thus, co-crystals have been reported with a metal complex, *i.e*. [Mn(1,10-phenanthroline)_3_][ClO_4_]_2_·(LH_2_) (Liu *et al.*, 1999 ▸). Monodentate coordination *via* a pyridyl-N atom was found in mononuclear HgI_2_(LH_2_)_2_ (Zeng *et al.*, 2008 ▸). Bidentate, bridging *via* both pyridyl-N atoms has been observed in binuclear {[Me_2_(4-HO_2_CC_6_H_4_CH_2_)Pt(4,4′-di-*t*-butyl-2,2′-bipyrid­yl]_2_(LH_2_)}_2_^2+^ (Fraser *et al.*, 2002 ▸) and in a polymeric silver salt, {AgBF_4_(LH_2_)·H_2_O}_*n*_ (Schauer *et al.*, 1998 ▸). In the analogous triflate salt {Ag_2_(O_3_SCF_3_)_2_(LH_2_)_3_}_*n*_ (Arman *et al.*, 2010 ▸), one LH_2_ bridges as in the BF_4_ salt (Schauer *et al.*, 1998 ▸) but the other two LH_2_ mol­ecules bridge one Ag^+^ *via* a pyridyl-N atom and another *via* the second pyridyl-N atom as well as a carbonyl-O atom, *i.e*. are tridentate. In a variation, tetra­dentate, bridging coordination *via* all four nitro­gen atoms is found in polymeric [CuL(**LH_2_**)(OH_2_]_*n*_ (Lloret *et al.*, 1989 ▸). Deprotonation of LH_2_ leads to a tetra­dentate ligand coordinating *via* all four nitro­gen atoms in PdL (Reger *et al.*, 2003 ▸). There are several examples of hexa­dentate-N_4_O_2_ coordination in copper(II) chemistry, as in the aforementioned [Cu**L**(LH_2_)(OH_2_]_*n*_ (Lloret *et al.*, 1989 ▸) and, for example, in polymeric [CuL(μ_2_-4,4′-bipyridyl-)(OH_2_)]_2_ (Zhang *et al.*, 2001 ▸).

## Synthesis and crystallization   

The di­amide (0.25 g), prepared in accord with the literature procedure (Schauer *et al.*, 1997 ▸), in ethanol (10 ml) was added to a ethanol solution (10 ml) of trimesic acid (Acros Organic, 0.18 g). The mixture was stirred for 2 h at room temperature. After standing for a few minutes, a white precipitate formed which was filtered off by vacuum suction. The filtrate was then left to stand under ambient conditions, yielding pale-yellow crystals after 2 weeks.

## Refinement   

Crystal data, data collection and structure refinement details are summarized in Table 7 ▸. The carbon-bound H atoms were placed in calculated positions (C—H = 0.95–0.99 Å) and were included in the refinement in the riding-model approximation, with *U*_iso_(H) set to 1.2*U*_eq_(C). The oxygen- and nitro­gen-bound H atoms were located in a difference Fourier map but were refined with distance restraints of O—H = 0.84±0.01 Å and N—H = 0.88±0.01 Å, and with *U*_iso_(H) set to 1.5*U*_eq_(O) and 1.2*U*_eq_(N).

## Supplementary Material

Crystal structure: contains datablock(s) I, global. DOI: 10.1107/S2056989016000980/hb7560sup1.cif

Structure factors: contains datablock(s) I. DOI: 10.1107/S2056989016000980/hb7560Isup2.hkl

Supporting information file. DOI: 10.1107/S2056989016000980/hb7560Isup3.cml

CCDC reference: 1447965

Additional supporting information:  crystallographic information; 3D view; checkCIF report

## Figures and Tables

**Table 1:** Selected geometric details (Å, °) for an *N*,*N*′-bis­(pyridin-2-ylmeth­yl)ethanedi­amide mol­ecule and protonated forms*^*a*^*

Coformer	C—N_py_—C	C_4_N_2_O_2_/N-ring	C(=O)—C(=O)	N_py_—C—C—N_amide_	Refcode*^*b*^*	Ref.
2-NH_2_C_6_H_4_CO_2_H*^*c*^*	119.01 (11)	69.63 (6)	1.54119 (16)	165.01 (10)	DIDZEX	Arman, Miller *et al.* (2012 ▸)
2,6-(NO_2_)_2_C_6_H_3_CO_2_^−^ *^*d*^*	123.00 (12)	72.92 (5)	1.5339 (18)	73.84 (15)	TIPHEH	Arman *et al.* (2013 ▸)
3,5-(CO_2_H)_2_C_6_H_3_CO_2_^−^	122.36 (18)	68.21 (8)	1.538 (3)	34.8 (2)	–	This work

Notes: (*a*) All di­amide mol­ecules/dianions are centrosymmetric; (*b*) Groom & Allen (2014 ▸); (*c*) 1:2 co-crystal with 2-amino­benzoic acid; (*d*) 1:2 salt with 2,6-di­nitro­benzoate in which both pyridyl-N atoms are protonated.

**Table 2:** Hydrogen-bond geometry (Å, °)

*D*—H⋯*A*	*D*—H	H⋯*A*	*D*⋯*A*	*D*—H⋯*A*
N2—H2*N*⋯O1^i^	0.88 (2)	2.38 (2)	2.704 (2)	102 (1)
O7—H7*O*⋯O6^ii^	0.85 (2)	1.77 (2)	2.614 (2)	178 (2)
O5—H5*O*⋯O2^iii^	0.85 (2)	1.69 (2)	2.5352 (19)	175 (2)
N2—H2*N*⋯O1^iv^	0.88 (2)	2.01 (2)	2.816 (2)	153 (2)
N1—H1*N*⋯O3^v^	0.89 (2)	1.73 (2)	2.604 (2)	169 (2)
C5—H5⋯O4^vi^	0.95	2.46	3.019 (3)	117
C6—H6*A*⋯O4^vi^	0.99	2.55	3.362 (3)	140
C2—H2⋯O2^i^	0.95	2.50	3.251 (3)	136
C3—H3⋯O6^vii^	0.95	2.59	3.068 (2)	112

Symmetry codes: (i) 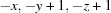; (ii) 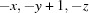; (iii) 
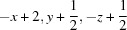
; (iv) 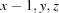; (v) 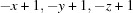; (vi) 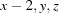; (vii) 
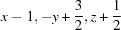
.

**Table 3:** Dihedral angles (°) for the 3,5-di­carb­oxy­benzoate anion in the title salt and in selected literature precedents*^*a*^*

Cation	C_6_/CO_2_	C_6_/CO_2_H	C_6_/CO_2_H	CSD Refcode*^*b*^*	Ref.
C_I*^*c*^*	8.6 (2)	4.96 (19)	12.82 (16)	QUFYIA	Santra *et al.* (2009 ▸)
	1.6 (2)	8.9 (2)	19.13 (15)		
C_II*^*c*^*	4.5 (3)	7.5 (4)	3.43 (18)	LUBJAV	Singh *et al.* (2015 ▸)
	2.1 (4)	2.0 (4)	2.6 (3)		
C_III	5.92 (11)	1.69 (14)	10.38 (10)	NIFGOY	Ferguson *et al.* (1998 ▸)
C_IV	25.13 (10)	22.50 (10)	11.60 (7)	CUMQUX	Basu *et al.* (2009 ▸)
dication	15.70 (13)	16.34 (12)	1.99 (10)	–	This work

Notes: (*a*) Refer to Scheme 2 for chemical structures; (*b*) Groom & Allen (2014 ▸); (*c*) Two independent anions.

**Table 4:** Short inter­atomic contacts (Å) in the title salt

Contact	Distance	Symmetry operation
C1⋯O1	3.096 (2)	−1 + *x*, *y*, *z*
C7⋯O3	3.072 (3)	1 − *x*, 1 − *y*, 1 − *z*
C11⋯O4	3.141 (3)	−1 + *x*, *y*, *z*
C14⋯H1*N*	2.74 (2)	1 − *x*, 1 − *y*, 1 − *z*
C10⋯H6*A*	2.77	1 + *x*, *y*, *z*
C14⋯H5*O*	2.631 (17)	-*x*, − 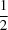 + *y*, 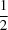 − *z*
C16⋯H7*O*	2.70 (2)	-*x*, 1 − *y*, −*z*

**Table 5:** Percentage contribution of the different inter­molecular inter­actions to the Hirshfeld surfaces for the dication, anion and salt

Contact	Dication	Anion	Salt
O⋯H/H⋯O	41.6	47.2	43.2
H⋯H	25.1	16.7	23.7
C⋯H/H⋯C	20.2	17.4	17.3
C⋯O/O⋯C	6.6	12.8	10.2
N⋯H/H⋯N	2.3	0.3	1.1
C⋯C	0.2	3.0	2.2
O⋯O	1.2	2.0	1.0
N⋯O/O⋯N	2.3	0.1	1.2
N⋯C/C⋯N	0.5	0.5	0.1

**Table 6:** Enrichment ratios (ER) for the dication, anion and salt

Contact	Dication	Anion	Salt
O⋯H/H⋯O	1.37	1.50	1.40
H⋯H	0.77	0.69	0.80
C⋯H/H⋯C	1.27	0.96	0.99
C⋯O/O⋯C	0.90	1.09	1.13
N⋯H/H⋯N	0.77	0.68	0.88
N⋯O/O⋯N	1.68	–	–

**Table 7:** Experimental details

Crystal data	
Chemical formula	C_14_H_16_N_4_O_2_^2+^·2C_9_H_5_O_6_^−^
*M*_r_	690.56
Crystal system, space group	Monoclinic, *P*2_1_/*c*
Temperature (K)	100
*a*, *b*, *c* (Å)	5.0436 (3), 18.4232 (10), 16.0796 (9)
β (°)	95.878 (5)
*V* (Å^3^)	1486.25 (15)
*Z*	2
Radiation type	Mo *K*α
μ (mm^−1^)	0.12
Crystal size (mm)	0.30 × 0.10 × 0.05

Data collection	
Diffractometer	Agilent SuperNova Dual diffractometer with an Atlas detector
Absorption correction	Multi-scan (*CrysAlis PRO*; Agilent, 2014 ▸)
*T*_min_, *T*_max_	0.580, 1.000
No. of measured, independent and observed [*I* > 2σ(*I*)] reflections	17686, 3410, 2656
*R*_int_	0.069
(sin θ/λ)_max_ (Å^−1^)	0.650

Refinement	
*R*[*F* ^2^ > 2σ(*F* ^2^)], *wR*(*F* ^2^), *S*	0.051, 0.134, 1.07
No. of reflections	3410
No. of parameters	238
No. of restraints	4
Δρ_max_, Δρ_min_ (e Å^−3^)	0.46, −0.26

Computer programs: *CrysAlis PRO* (Agilent, 2014 ▸), *SHELXS97* (Sheldrick, 2008 ▸), *SHELXL2014* (Sheldrick, 2015 ▸), *ORTEP-3 for Windows* (Farrugia, 2012 ▸), *QMol* (Gans & Shalloway, 2001 ▸), *DIAMOND* (Brandenburg, 2006 ▸) and *publCIF* (Westrip, 2010 ▸).

## supplementary crystallographic information

### Crystal data

**Table:**

C_14_H_16_N_4_O_2_^2+^·2C_9_H_5_O_6_^−^	*F*(000) = 716
*M**_r_* = 690.56	*D*_x_ = 1.543 Mg m^−^^3^
Monoclinic, *P*2_1_/*c*	Mo *K*α radiation, λ = 0.71073 Å
*a* = 5.0436 (3) Å	Cell parameters from 6152 reflections
*b* = 18.4232 (10) Å	θ = 3.4–29.2°
*c* = 16.0796 (9) Å	µ = 0.12 mm^−^^1^
β = 95.878 (5)°	*T* = 100 K
*V* = 1486.25 (15) Å^3^	Prism, pale-yellow
*Z* = 2	0.30 × 0.10 × 0.05 mm

### Data collection

**Table:**

Agilent SuperNova Dual diffractometer with an Atlas detector	3410 independent reflections
Radiation source: SuperNova (Mo) X-ray Source	2656 reflections with *I* > 2σ(*I*)
Mirror monochromator	*R*_int_ = 0.069
Detector resolution: 10.4041 pixels mm^-1^	θ_max_ = 27.5°, θ_min_ = 3.4°
ω scan	*h* = −6→6
Absorption correction: multi-scan (*CrysAlis PRO*; Agilent, 2014)	*k* = −23→23
*T*_min_ = 0.580, *T*_max_ = 1.000	*l* = −20→20
17686 measured reflections	

### Refinement

**Table:**

Refinement on *F*^2^	4 restraints
Least-squares matrix: full	Hydrogen site location: mixed
*R*[*F*^2^ > 2σ(*F*^2^)] = 0.051	*w* = 1/[σ^2^(*F*_o_^2^) + (0.0563*P*)^2^ + 0.8519*P*] where *P* = (*F*_o_^2^ + 2*F*_c_^2^)/3
*wR*(*F*^2^) = 0.134	(Δ/σ)_max_ < 0.001
*S* = 1.07	Δρ_max_ = 0.46 e Å^−^^3^
3410 reflections	Δρ_min_ = −0.26 e Å^−^^3^
238 parameters	

### Special details

**Table:**

Geometry. All esds (except the esd in the dihedral angle between two l.s. planes) are estimated using the full covariance matrix. The cell esds are taken into account individually in the estimation of esds in distances, angles and torsion angles; correlations between esds in cell parameters are only used when they are defined by crystal symmetry. An approximate (isotropic) treatment of cell esds is used for estimating esds involving l.s. planes.

### Fractional atomic coordinates and isotropic or equivalent isotropic displacement parameters (Å^2^)

**Table:**

	*x*	*y*	*z*	*U*_iso_*/*U*_eq_	
O1	0.2441 (3)	0.56058 (7)	0.47153 (9)	0.0231 (3)	
N1	−0.3956 (3)	0.69095 (9)	0.53385 (11)	0.0198 (4)	
H1N	−0.285 (4)	0.6629 (11)	0.5665 (12)	0.024*	
N2	−0.2089 (3)	0.56875 (9)	0.45086 (11)	0.0194 (4)	
H2N	−0.364 (3)	0.5516 (12)	0.4615 (14)	0.023*	
C1	−0.3894 (4)	0.69266 (10)	0.45027 (12)	0.0187 (4)	
C2	−0.5582 (4)	0.73355 (11)	0.57322 (13)	0.0226 (4)	
H2	−0.5589	0.7300	0.6321	0.027*	
C3	−0.7242 (4)	0.78235 (11)	0.52887 (13)	0.0241 (4)	
H3	−0.8446	0.8113	0.5562	0.029*	
C4	−0.7117 (4)	0.78821 (11)	0.44357 (13)	0.0234 (4)	
H4	−0.8184	0.8231	0.4122	0.028*	
C5	−0.5438 (4)	0.74330 (10)	0.40389 (13)	0.0209 (4)	
H5	−0.5349	0.7472	0.3453	0.025*	
C6	−0.2190 (4)	0.63885 (10)	0.40966 (13)	0.0208 (4)	
H6A	−0.2906	0.6325	0.3504	0.025*	
H6B	−0.0358	0.6584	0.4107	0.025*	
C7	0.0204 (4)	0.53666 (11)	0.47870 (12)	0.0197 (4)	
O2	0.8690 (3)	0.32072 (7)	0.27064 (9)	0.0253 (3)	
O3	1.1233 (3)	0.39299 (8)	0.35861 (9)	0.0298 (4)	
O4	1.2729 (3)	0.64690 (8)	0.25738 (10)	0.0260 (3)	
O5	0.9119 (3)	0.69980 (7)	0.19086 (9)	0.0243 (3)	
H5O	0.994 (5)	0.7391 (9)	0.2034 (16)	0.036*	
O6	0.2374 (3)	0.55161 (7)	0.03570 (9)	0.0220 (3)	
O7	0.1837 (3)	0.43588 (7)	0.07250 (9)	0.0217 (3)	
H7O	0.049 (3)	0.4407 (14)	0.0370 (13)	0.033*	
C8	0.8550 (4)	0.44714 (10)	0.24689 (12)	0.0183 (4)	
C9	0.9905 (4)	0.51294 (10)	0.25715 (12)	0.0178 (4)	
H9	1.1439	0.5167	0.2964	0.021*	
C10	0.9018 (4)	0.57340 (10)	0.20997 (12)	0.0171 (4)	
C11	0.6784 (4)	0.56752 (10)	0.15260 (12)	0.0180 (4)	
H11	0.6170	0.6086	0.1205	0.022*	
C12	0.5438 (4)	0.50196 (10)	0.14184 (12)	0.0178 (4)	
C13	0.6305 (4)	0.44181 (10)	0.18970 (12)	0.0180 (4)	
H13	0.5360	0.3972	0.1832	0.022*	
C14	0.9579 (4)	0.38131 (10)	0.29671 (12)	0.0197 (4)	
C15	1.0500 (4)	0.64353 (10)	0.22231 (12)	0.0191 (4)	
C16	0.3081 (4)	0.49865 (10)	0.07891 (12)	0.0184 (4)	

### Atomic displacement parameters (Å^2^)

**Table:**

	*U*^11^	*U*^22^	*U*^33^	*U*^12^	*U*^13^	*U*^23^
O1	0.0151 (7)	0.0200 (7)	0.0337 (8)	−0.0005 (5)	−0.0001 (6)	0.0019 (6)
N1	0.0211 (9)	0.0166 (8)	0.0204 (9)	−0.0001 (6)	−0.0033 (7)	0.0019 (7)
N2	0.0166 (8)	0.0145 (8)	0.0264 (9)	0.0000 (6)	−0.0006 (7)	0.0020 (7)
C1	0.0193 (9)	0.0160 (9)	0.0197 (10)	−0.0030 (7)	−0.0031 (7)	0.0000 (8)
C2	0.0266 (11)	0.0206 (10)	0.0197 (10)	−0.0036 (8)	−0.0014 (8)	−0.0006 (8)
C3	0.0276 (11)	0.0176 (10)	0.0272 (11)	−0.0007 (8)	0.0027 (8)	−0.0034 (8)
C4	0.0274 (11)	0.0145 (9)	0.0270 (11)	0.0008 (8)	−0.0032 (8)	0.0008 (8)
C5	0.0252 (10)	0.0166 (9)	0.0201 (10)	−0.0018 (8)	−0.0024 (8)	0.0006 (8)
C6	0.0221 (10)	0.0176 (10)	0.0221 (10)	−0.0002 (7)	−0.0006 (8)	0.0021 (8)
C7	0.0188 (9)	0.0191 (10)	0.0207 (10)	−0.0003 (7)	−0.0002 (7)	−0.0034 (8)
O2	0.0290 (8)	0.0153 (7)	0.0298 (8)	0.0007 (6)	−0.0060 (6)	0.0020 (6)
O3	0.0359 (9)	0.0218 (8)	0.0281 (8)	0.0010 (6)	−0.0140 (7)	0.0031 (6)
O4	0.0214 (7)	0.0204 (7)	0.0341 (9)	−0.0014 (6)	−0.0071 (6)	−0.0030 (6)
O5	0.0260 (8)	0.0135 (7)	0.0313 (8)	−0.0026 (6)	−0.0074 (6)	0.0017 (6)
O6	0.0220 (7)	0.0185 (7)	0.0234 (7)	−0.0015 (5)	−0.0072 (6)	0.0036 (6)
O7	0.0210 (7)	0.0163 (7)	0.0256 (8)	−0.0035 (5)	−0.0084 (6)	0.0020 (6)
C8	0.0220 (10)	0.0154 (9)	0.0171 (9)	0.0028 (7)	0.0009 (7)	0.0000 (7)
C9	0.0186 (9)	0.0192 (9)	0.0149 (9)	0.0011 (7)	−0.0012 (7)	−0.0019 (7)
C10	0.0178 (9)	0.0148 (9)	0.0185 (9)	−0.0003 (7)	0.0016 (7)	−0.0013 (7)
C11	0.0204 (10)	0.0146 (9)	0.0185 (10)	0.0037 (7)	−0.0004 (8)	0.0013 (7)
C12	0.0175 (9)	0.0169 (9)	0.0184 (10)	0.0010 (7)	−0.0001 (7)	0.0001 (7)
C13	0.0194 (9)	0.0150 (9)	0.0196 (10)	0.0000 (7)	0.0013 (7)	−0.0018 (7)
C14	0.0206 (9)	0.0164 (9)	0.0214 (10)	0.0021 (7)	−0.0010 (8)	0.0016 (8)
C15	0.0226 (10)	0.0166 (9)	0.0176 (9)	−0.0001 (7)	0.0000 (8)	−0.0015 (7)
C16	0.0199 (10)	0.0162 (9)	0.0186 (10)	−0.0001 (7)	−0.0001 (8)	0.0002 (7)

### Geometric parameters (Å, º)

**Table:**

O1—C7	1.227 (2)	O3—C14	1.250 (2)
N1—C2	1.340 (3)	O4—C15	1.206 (2)
N1—C1	1.348 (3)	O5—C15	1.320 (2)
N1—H1N	0.892 (10)	O5—H5O	0.848 (10)
N2—C7	1.335 (3)	O6—C16	1.229 (2)
N2—C6	1.450 (2)	O7—C16	1.315 (2)
N2—H2N	0.878 (10)	O7—H7O	0.847 (10)
C1—C5	1.384 (3)	C8—C13	1.387 (3)
C1—C6	1.504 (3)	C8—C9	1.393 (3)
C2—C3	1.377 (3)	C8—C14	1.516 (3)
C2—H2	0.9500	C9—C10	1.395 (3)
C3—C4	1.384 (3)	C9—H9	0.9500
C3—H3	0.9500	C10—C11	1.385 (3)
C4—C5	1.385 (3)	C10—C15	1.496 (3)
C4—H4	0.9500	C11—C12	1.388 (3)
C5—H5	0.9500	C11—H11	0.9500
C6—H6A	0.9900	C12—C13	1.394 (3)
C6—H6B	0.9900	C12—C16	1.481 (3)
C7—C7^i^	1.538 (4)	C13—H13	0.9500
O2—C14	1.259 (2)		
			
C2—N1—C1	122.36 (17)	C15—O5—H5O	110.7 (18)
C2—N1—H1N	116.1 (15)	C16—O7—H7O	107.8 (17)
C1—N1—H1N	121.5 (15)	C13—C8—C9	119.82 (17)
C7—N2—C6	122.43 (17)	C13—C8—C14	120.39 (17)
C7—N2—H2N	122.4 (15)	C9—C8—C14	119.77 (17)
C6—N2—H2N	114.8 (15)	C8—C9—C10	120.28 (17)
N1—C1—C5	118.93 (18)	C8—C9—H9	119.9
N1—C1—C6	119.35 (17)	C10—C9—H9	119.9
C5—C1—C6	121.71 (18)	C11—C10—C9	119.56 (17)
N1—C2—C3	120.45 (19)	C11—C10—C15	121.15 (17)
N1—C2—H2	119.8	C9—C10—C15	119.29 (17)
C3—C2—H2	119.8	C10—C11—C12	120.34 (17)
C2—C3—C4	118.52 (19)	C10—C11—H11	119.8
C2—C3—H3	120.7	C12—C11—H11	119.8
C4—C3—H3	120.7	C11—C12—C13	120.10 (17)
C3—C4—C5	120.13 (19)	C11—C12—C16	117.97 (16)
C3—C4—H4	119.9	C13—C12—C16	121.92 (17)
C5—C4—H4	119.9	C8—C13—C12	119.89 (17)
C1—C5—C4	119.44 (19)	C8—C13—H13	120.1
C1—C5—H5	120.3	C12—C13—H13	120.1
C4—C5—H5	120.3	O3—C14—O2	127.13 (18)
N2—C6—C1	112.55 (17)	O3—C14—C8	116.59 (17)
N2—C6—H6A	109.1	O2—C14—C8	116.27 (17)
C1—C6—H6A	109.1	O4—C15—O5	124.63 (17)
N2—C6—H6B	109.1	O4—C15—C10	122.39 (17)
C1—C6—H6B	109.1	O5—C15—C10	112.98 (16)
H6A—C6—H6B	107.8	O6—C16—O7	123.05 (17)
O1—C7—N2	125.63 (19)	O6—C16—C12	121.29 (17)
O1—C7—C7^i^	121.6 (2)	O7—C16—C12	115.66 (16)
N2—C7—C7^i^	112.8 (2)		
			
C2—N1—C1—C5	4.2 (3)	C10—C11—C12—C13	1.0 (3)
C2—N1—C1—C6	−174.80 (18)	C10—C11—C12—C16	−179.31 (17)
C1—N1—C2—C3	−1.2 (3)	C9—C8—C13—C12	1.0 (3)
N1—C2—C3—C4	−2.4 (3)	C14—C8—C13—C12	−177.46 (18)
C2—C3—C4—C5	2.9 (3)	C11—C12—C13—C8	−1.4 (3)
N1—C1—C5—C4	−3.5 (3)	C16—C12—C13—C8	178.90 (18)
C6—C1—C5—C4	175.42 (18)	C13—C8—C14—O3	−165.39 (18)
C3—C4—C5—C1	0.0 (3)	C9—C8—C14—O3	16.1 (3)
C7—N2—C6—C1	−125.7 (2)	C13—C8—C14—O2	15.3 (3)
N1—C1—C6—N2	34.8 (2)	C9—C8—C14—O2	−163.19 (18)
C5—C1—C6—N2	−144.13 (19)	C11—C10—C15—O4	−163.64 (19)
C6—N2—C7—O1	−1.8 (3)	C9—C10—C15—O4	16.4 (3)
C6—N2—C7—C7^i^	179.1 (2)	C11—C10—C15—O5	16.2 (3)
C13—C8—C9—C10	−0.2 (3)	C9—C10—C15—O5	−163.84 (17)
C14—C8—C9—C10	178.26 (17)	C11—C12—C16—O6	2.0 (3)
C8—C9—C10—C11	−0.2 (3)	C13—C12—C16—O6	−178.26 (18)
C8—C9—C10—C15	179.80 (17)	C11—C12—C16—O7	−178.40 (17)
C9—C10—C11—C12	−0.2 (3)	C13—C12—C16—O7	1.3 (3)
C15—C10—C11—C12	179.82 (18)		

Symmetry code: (i) −*x*, −*y*+1, −*z*+1.

### Hydrogen-bond geometry (Å, º)

**Table:**

*D*—H···*A*	*D*—H	H···*A*	*D*···*A*	*D*—H···*A*
N2—H2*N*···O1^i^	0.88 (2)	2.38 (2)	2.704 (2)	102 (1)
O7—H7*O*···O6^ii^	0.85 (2)	1.77 (2)	2.614 (2)	178 (2)
O5—H5*O*···O2^iii^	0.85 (2)	1.69 (2)	2.5352 (19)	175 (2)
N2—H2*N*···O1^iv^	0.88 (2)	2.01 (2)	2.816 (2)	153 (2)
N1—H1*N*···O3^v^	0.89 (2)	1.73 (2)	2.604 (2)	169 (2)
C5—H5···O4^vi^	0.95	2.46	3.019 (3)	117
C6—H6*A*···O4^vi^	0.99	2.55	3.362 (3)	140
C2—H2···O2^i^	0.95	2.50	3.251 (3)	136
C3—H3···O6^vii^	0.95	2.59	3.068 (2)	112

Symmetry codes: (i) −*x*, −*y*+1, −*z*+1; (ii) −*x*, −*y*+1, −*z*; (iii) −*x*+2, *y*+1/2, −*z*+1/2; (iv) *x*−1, *y*, *z*; (v) −*x*+1, −*y*+1, −*z*+1; (vi) *x*−2, *y*, *z*; (vii) *x*−1, −*y*+3/2, *z*+1/2.
